# Screening and identification of the core immune‐related genes and immune cell infiltration in severe burns and sepsis

**DOI:** 10.1111/jcmm.17749

**Published:** 2023-04-15

**Authors:** Wenxing Su, Wei Li, Yuanyuan Zhang, Kuan Wang, Maolin Chen, Xiaoming Chen, Dazhuang Li, Ping Zhang, Daojiang Yu

**Affiliations:** ^1^ Department of Plastic and Burns Surgery The Second Affiliated Hospital of Chengdu Medical College, China National Nuclear Corporation 416 Hospital Chengdu China; ^2^ Department of Urology The First Affiliated Hospital of Chengdu Medical College Chengdu China; ^3^ Department of Medical Laboratory Xindu District People's Hospital of Chengdu Chengdu China; ^4^ Department of Cosmetic Plastic and Burns Surgery The First Affiliated Hospital of Chengdu Medical College Chengdu China; ^5^ Department of Orthopedics The Affiliated Hospital of Yangzhou University, Yangzhou University Yangzhou China

**Keywords:** burns, core immune‐related genes, differentially expressed genes, immune cell infiltration, microarray data, sepsis

## Abstract

Severe burns often have a high mortality rate due to sepsis, but the genetic and immune crosstalk between them remains unclear. In the present study, the GSE77791 and GSE95233 datasets were analysed to identify immune‐related differentially expressed genes (DEGs) involved in disease progression in both burns and sepsis. Subsequently, weighted gene coexpression network analysis (WGCNA), gene enrichment analysis, protein–protein interaction (PPI) network construction, immune cell infiltration analysis, core gene identification, coexpression network analysis and clinical correlation analysis were performed. A total of 282 common DEGs associated with burns and sepsis were identified. Kyoto Encyclopedia of Genes and Genomes pathway analysis identified the following enriched pathways in burns and sepsis: metabolic pathways; complement and coagulation cascades; legionellosis; starch and sucrose metabolism; and ferroptosis. Finally, six core DEGs were identified, namely, IL10, RETN, THBS1, FGF13, LCN2 and MMP9. Correlation analysis showed that some core DEGs were significantly associated with simultaneous dysregulation of immune cells. Of these, RETN upregulation was associated with a worse prognosis. The immune‐related genes and dysregulated immune cells in severe burns and sepsis provide potential research directions for diagnosis and treatment.

## 1 INTRODUCTION

Burns are the fourth most common trauma worldwide,^1^ and they often lead to sepsis and high mortality. Sepsis is an excessive immune response induced by severe infection that can trigger systemic multiple organ dysfunction and ultimately endanger the patient's life.^2^ After sepsis occurs in the body, the production of proinflammatory cytokines increases, the number of lymphocytes decreases, and immune function is suppressed,^3^ leading to dysfunction of blood vessels, nerves, hormones, metabolism and coagulation.^4^, ^5^

In severe burns, the adaptive immune function represented by T cells is usually inhibited, and the inflammatory response represented by neutrophils and dendritic cells is activated.^6^ The disordered immune state causes the body to easily induce severe infection. Inflammatory factors, cytokines and immune cells have significant prognostic value in severe burns.^7^ Numerous studies have shown that patients with burn‐induced sepsis are characterized by opportunistic infections and immunosuppression.^3^, ^8^, ^9^, ^10^, ^11^ The mechanisms of sepsis‐induced immunosuppression include apoptosis depletion of immune cells, increased expression of negative costimulatory molecules, increased expression of regulatory T cells (Tregs) and programmed cell death of CD4^+^ T cells.^3^ In the early stage of sepsis, macrophages and neutrophils initiate a proinflammatory cascade, which is mainly achieved by the release of cytokines, such as tumour necrosis factor (TNF‐α) and interleukin (IL1).^11^ In addition, anti‐inflammatory mediators, such as IL4 and IL10, are also released during sepsis, a process that not only suppresses the proinflammatory cascade but also leads to a state of relative immunosuppression in patients with sepsis.^12^ In the late stage of sepsis, excessive apoptosis of macrophages leads to immune dysfunction and organ damage,^13^, ^14^ which increases the probability of secondary host infection and eventually leads to patient death.

Currently, there are no tests or diagnostic criteria to identify sepsis in critically ill patients. Although many patients are affected by burn sepsis, the treatment of burn sepsis still faces many difficulties due to the lack of clear diagnosis and treatment guidelines. To overcome the delay of diagnosis and treatment, we used weighted gene coexpression network analysis (WGCNA) to explore the correlation between module signature genes (highly interconnected gene set) and the occurrence of sepsis and burns. At the same time, DEGs of burns and sepsis were obtained with GEO2R. The genes of the overlapping module genes and DEGs were used for further analysis. Subsequently, core gene identification, immune infiltration analysis and clinical relevance studies were performed to explore the potential link between burns and sepsis, thus providing new references for the diagnosis and treatment of patients with secondary sepsis after burns.

## 2 METHODS

### 2.1 Raw data

The datasets of GSE95233^15^ and GSE77791^15^ (GPL570 platform, Affymetrix Human Genome U133 Plus 2.0 Array) were downloaded from the Gene Expression Omnibus (GEO) database (http://www.ncbi.nlm.nih.gov/geo).^16^ The GSE95233 dataset contains 51 sepsis patients and 22 healthy volunteers. We selected blood samples without any treatment on the first day for comparative analysis. The GSE77791 dataset contains 30 severely burned patients (a total burns surface area (TBSA) range from 30% to 98%) and 13 healthy volunteers. Similarly, we selected pre‐treatment blood samples for subsequent analysis. Some research methods in this paper have been applied in our previously published manuscripts.^17^

### 2.2 WGCNA network construction and module identification

Coexpression networks were constructed to identify coexpression modules via the WGCNA package in R.^18^ First, the samples were subjected to hierarchical clustering analysis to assess whether there were any significant outliers; second, the soft threshold power β was calculated by the picksoftworthold algorithm in R to construct a biologically meaningful scale‐free network; third, the topological overlap matrix establishes network interconnectivity and identifies gene modules based on a dynamic tree‐cutting algorithm; fourth, modules were linked to clinical features by calculating gene significance and module membership. The genes involved in the corresponding modules were used for subsequent analysis. Finally, the eigengene network was visualized.

### 2.3 Identification of common DEGs

GEO2R (www.ncbi.nlm.nih.gov/geo/ge2r) is an online tool based on Limma package for analysing DEGs.^19^ ‘Adjusted *p*‐values < 0.01 and |logFC| ≥ 1’ were defined as the thresholds for the screening of DEGs. We extracted the genes in the WGCNA modules most positively associated with sepsis and burns and cross‐analysed these two gene lists to obtain common DEGs.

### 2.4 Enrichment analyses of common DEGs

To further understand which biological functions these common DEGs are involved in, we performed gene enrichment analysis using the KOBAS 3.0 database, including gene ontology (GO) and Kyoto Encyclopedia of Genes and Genomes (KEGG) pathways.^20^ The species was set as Homo sapiens. Adjusted *p*‐values < 0.05 was considered significant.

### 2.5 PPI network construction and identification of core immune‐related DEGs

We constructed the PPI network corresponding to common DEGs from the STRING database (STRING; http://string‐db.org) (version 11.5)^21^ and visualized it with Cytoscape software (http://www.cytoscape.org) (version 3.9.0).^22^ Subsequently, we identified core functional modules through Cytoscape's plug‐in molecular complex detection technology (MCODE).^23^ Set the selection criteria as: K‐core = 2, degree cutoff = 2, max depth = 100, and node score cutoff = 0.2. We downloaded the currently known immune‐related genes from the Immunology Database and Analysis Portal database (ImmPort; https://www.immport.org) (Table S1). Then, Venn diagrams were intersected to obtain core immune‐related DEGs in sepsis and burns. Finally, we constructed a co‐expression network of core immune‐related DEGs using the GeneMANIA database (http://www.genemania.org/).^24^

### 2.6 Immune infiltration analyses

We assessed the corresponding immune cell infiltration status of these two datasets by Immune Cell Abundance Identifier (ImmuCellAI) (http://bioinfo.life.hust.edu.cn/ImmuCellAI/#!/),^25^ which provides comprehensive predictions of immune cell abundance by assessing the abundance of 24 immune cell types. We further compared the differences between the disease group and the control group by *t*‐test. Finally, we explored the connection between immune cells and core immune‐related DEGs by Spearman correlation analysis.

### 2.7 Clinical significance of core immune‐related DEGs in sepsis and burns

To verify our results, the expression of core immune‐related DEGs was extracted from GSE95233, GSE77791, GSE19743 and GSE57065, and the difference between disease group and normal group was analysed by *t*‐test. Receiver operating characteristic curve analysis was used to examine the diagnostic value of core immune‐related DEGs. Since there is survival information for the burns and sepsis cohort in the GSE19743 and GSE95233, we compared the differences in core immune‐related DEGs between the survival and non‐survival groups.

## 3 RESULTS

### 3.1 WGCNA network construction and module identification

**FIGURE 1:**
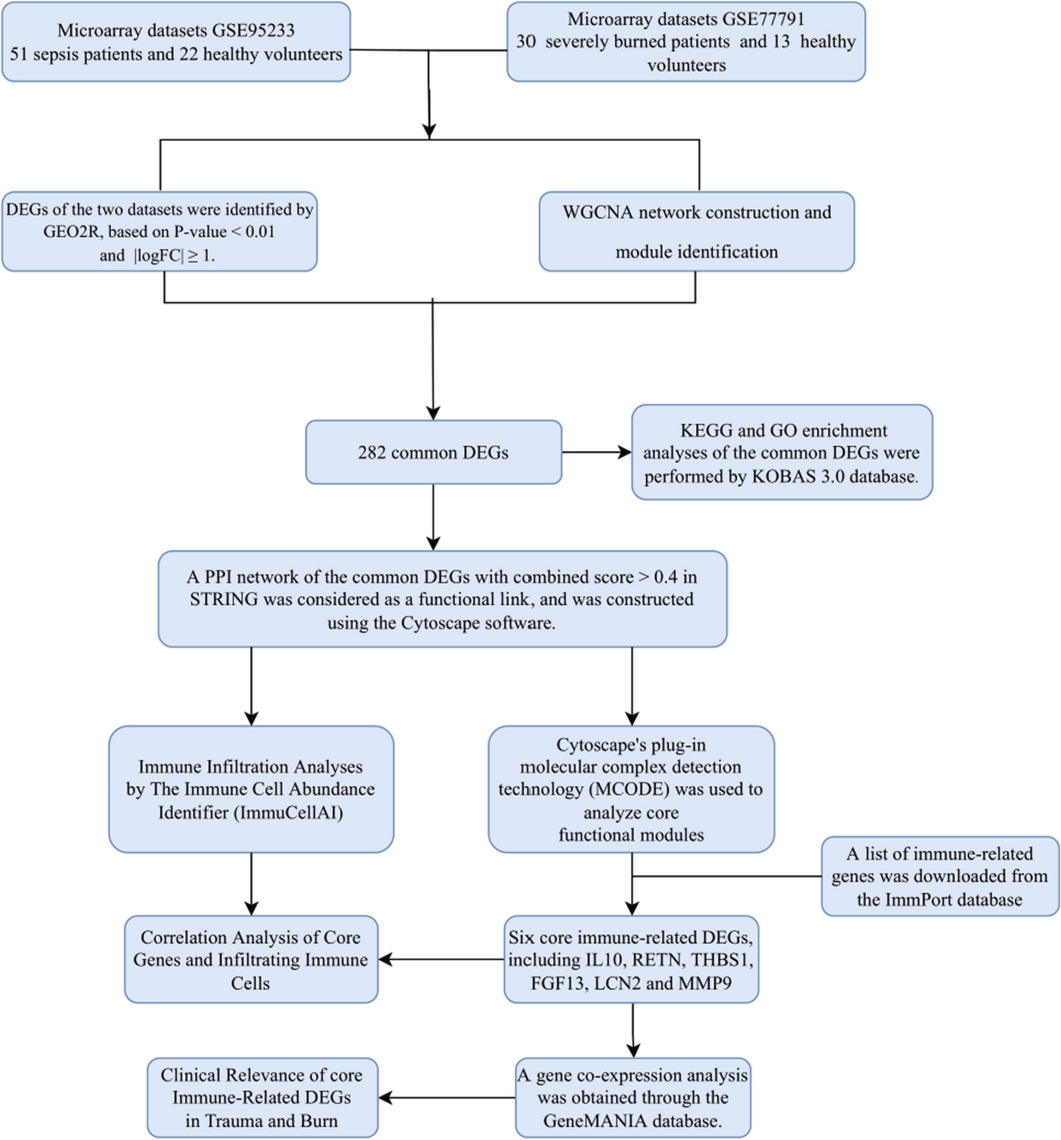
Research design flow chart. In this study, we used WGCNA to explore the correlation between module signature genes and the occurrence of sepsis and burns. At the same time, differentially expressed genes (DEGs) of burns and sepsis were obtained with GEO2R. The genes of the overlapping part between the module genes and DEGs were used for further analysis. Subsequently, core gene identification, immune infiltration analysis and clinical relevance studies were performed to explore the potential link between burns and sepsis, thus providing new references for the diagnosis and treatment of patients with secondary sepsis after burns.

**FIGURE 2:**
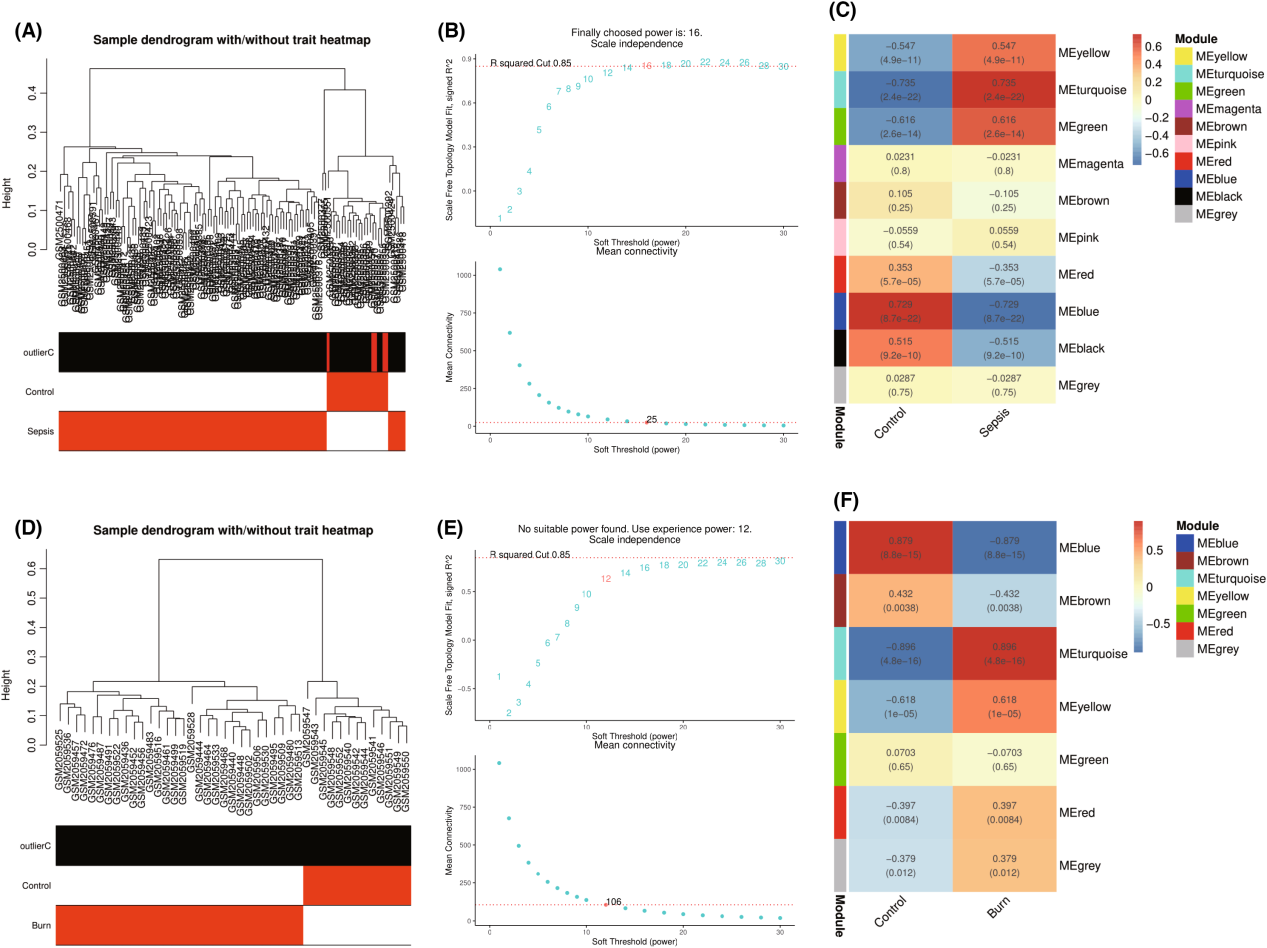
(A,D) Clustering dendrogram of samples based on their Euclidean distance in GSE95233 and GSE77791. (B,E) Determination of soft‐thresholding power for GSE95233 and GSE77791. (C,F) Heatmap of the correlation between module eigengenes and the occurrence of sepsis and burns. Modules with different colours represent different gene modules, and the numbers in the modules represent the correlation between the module and the phenotype.

The flowchart of the present study is shown in Figure 1. WGCNA was used to explore core modules significantly associated with disease. Potential outlier samples marked with red bars in the <outlierC> row were eliminated in the GSE95233 dataset, and no samples were eliminated in the GSE77791 dataset (Figure 2A,D). In the WGCNA method, *b* = 16 was the optimal soft threshold for the GSE95233 dataset (Figure 2B), and *b* = 12 was the optimal soft threshold for the GSE77791 dataset (Figure 2E). Ten and seven modules were identified in the GSE95233 and GSE77791 datasets, respectively. Correlation analysis showed that the turquoise module was strongly positively correlated with sepsis (*r* = 0.735), while the blue module was strongly negatively correlated with sepsis (*r* = −0.729) (Figure 2C). For burns, the turquoise module showed a strong positive correlation (*r* = 0.896), while the blue module had a strong negative correlation (*r* = −0.879) (Figure 2F).

### 3.2 Identification of common DEGs

**FIGURE 3:**
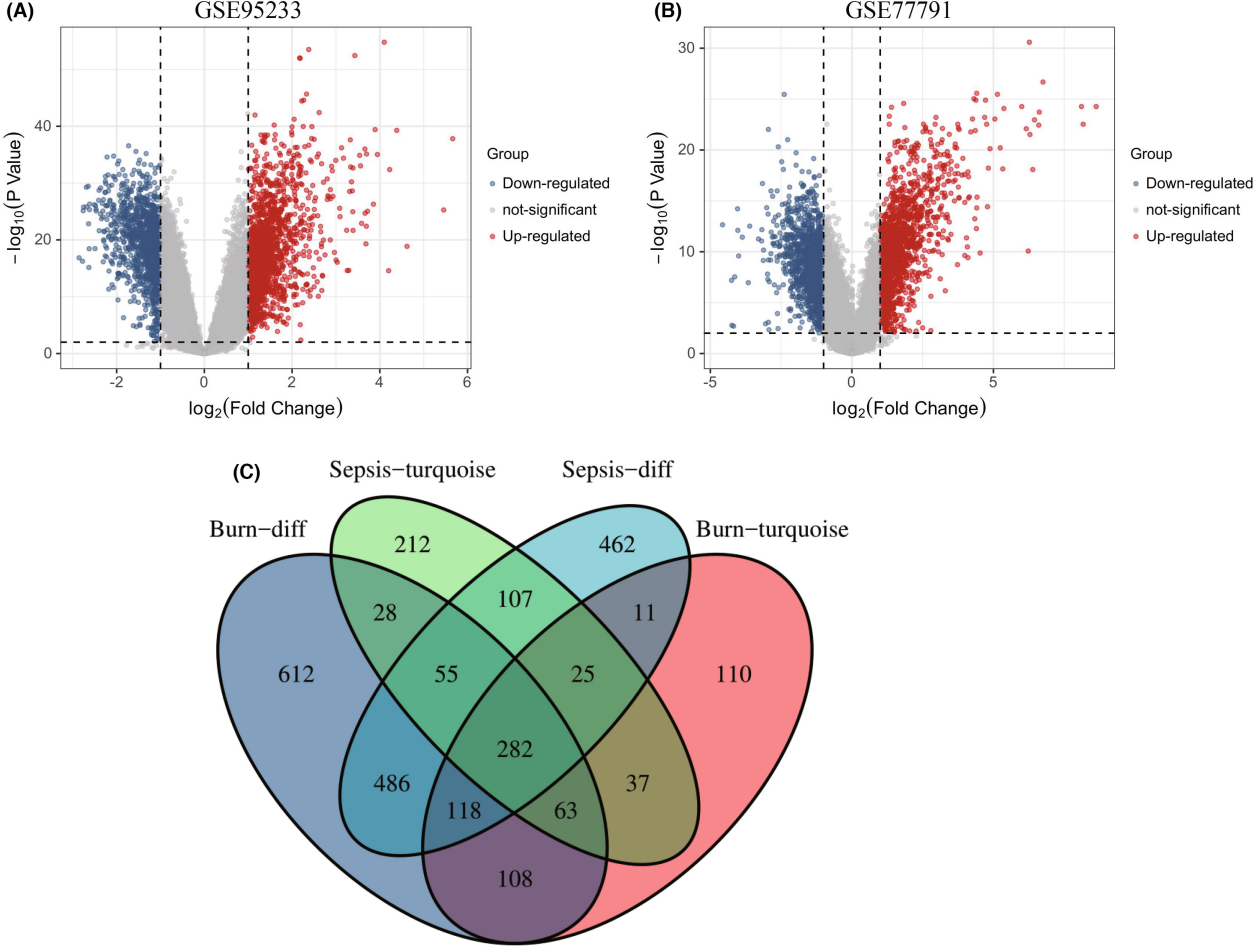
(A) The volcano map of GSE95233. (B) The volcano map of GSE77791. Red dots represent up‐regulated genes, blue dots represent down‐regulated genes and grey dots represent genes with no significant difference. (C) Venn diagram show that 282 common differentially expressed genes (DEGs) in GSE95233 and GSE77791.

According to the GEO2R online analysis results, 1546 DEGs and 1752 DEGs were identified in the GSE95233 and GSE77791 datasets, respectively (Figure 3A,B). In addition, we extracted genes from the most prominent modules for burns and sepsis. As shown in the flow chart, we used WGCNA to obtain the module signature genes closely related to diseases. The module signature genes were then intersected with the DEGs identified by GEO2R to further obtain the common DEGs. Through Venn diagram calculation combined with WGCNA, we obtained 282 common DEGs between sepsis and burns (Figure 3C and Table S2).

### 3.3 Analysis of the functional characteristics

**FIGURE 4:**
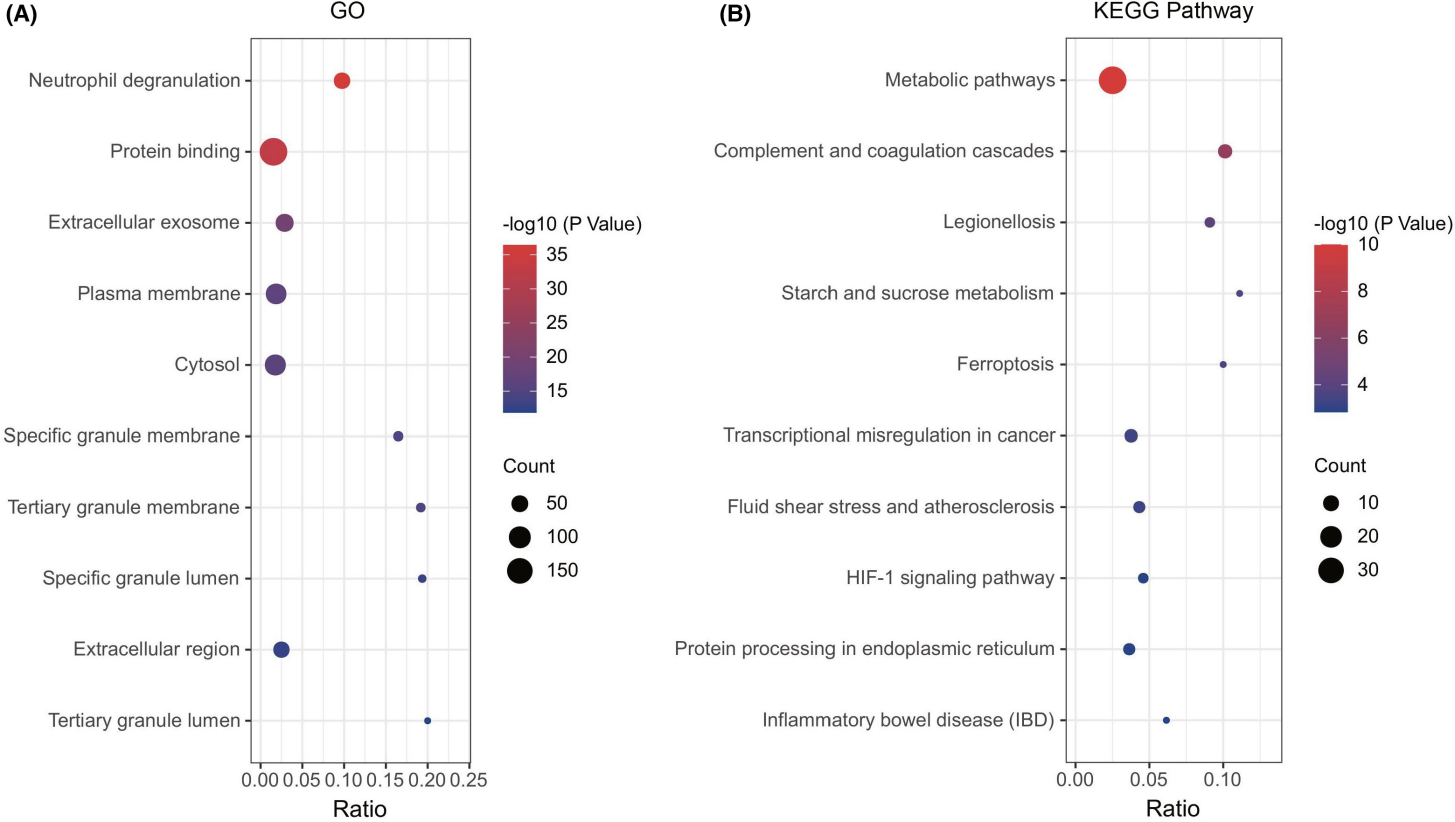
(A) Enrichment result of common DEGs GO term; (B) enrichment result of common differentially expressed genes (DEGs) KEGG pathway. Adjusted *p*‐value < 0.05 was considered significant. The ordinate represents the enriched term, and the abscissa represents the proportion of genes involved in the term. The size of the dots represents the number of genes, and the colour of the dots represents the *p* value.

GO analysis showed that the common DEGs were significantly enriched in neutrophil degranulation, protein binding, extracellular exosome, plasma membrane and cytosol (Figure 4A). KEGG pathway analysis showed that the DEGs were mainly concentrated in the following pathways: metabolic pathways; complement and coagulation cascades; legionellosis; starch and sucrose metabolism; and ferroptosis (Figure 4B).

### 3.4 Protein‐protein interaction (PPI) network construction and analysis of core immune‐related DEGs

**FIGURE 5:**
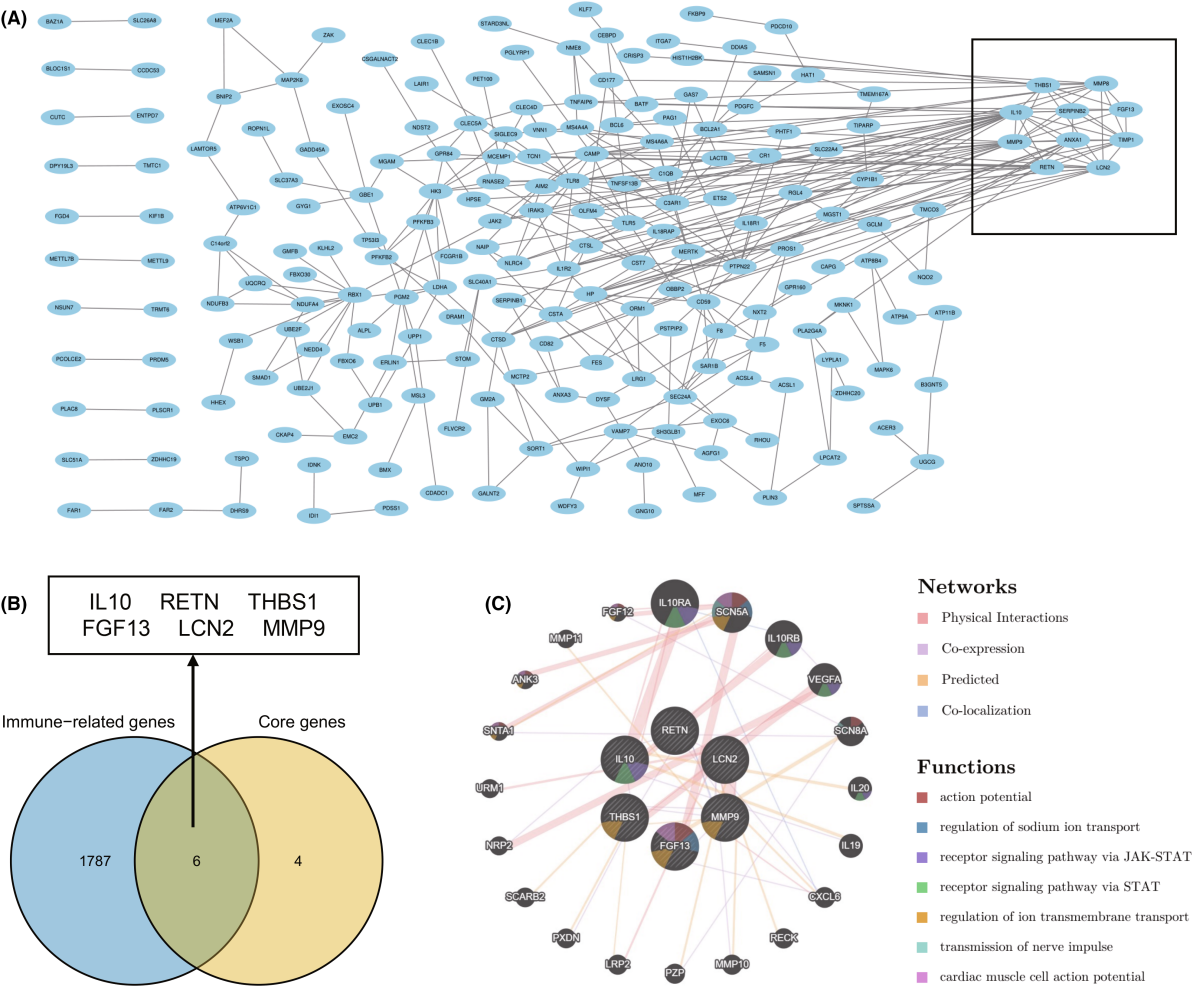
(A) PPI network constructed using the STRING database. The modules in the box are the core genes screened out by the MCODE algorithm. (B) The core module genes were intersected with immune‐related genes from the database to obtain six core immune‐related differentially expressed genes (DEGs). (C) Core immune‐related DEGs and their co‐expression genes were analysed via GeneMANIA.

**FIGURE 6:**
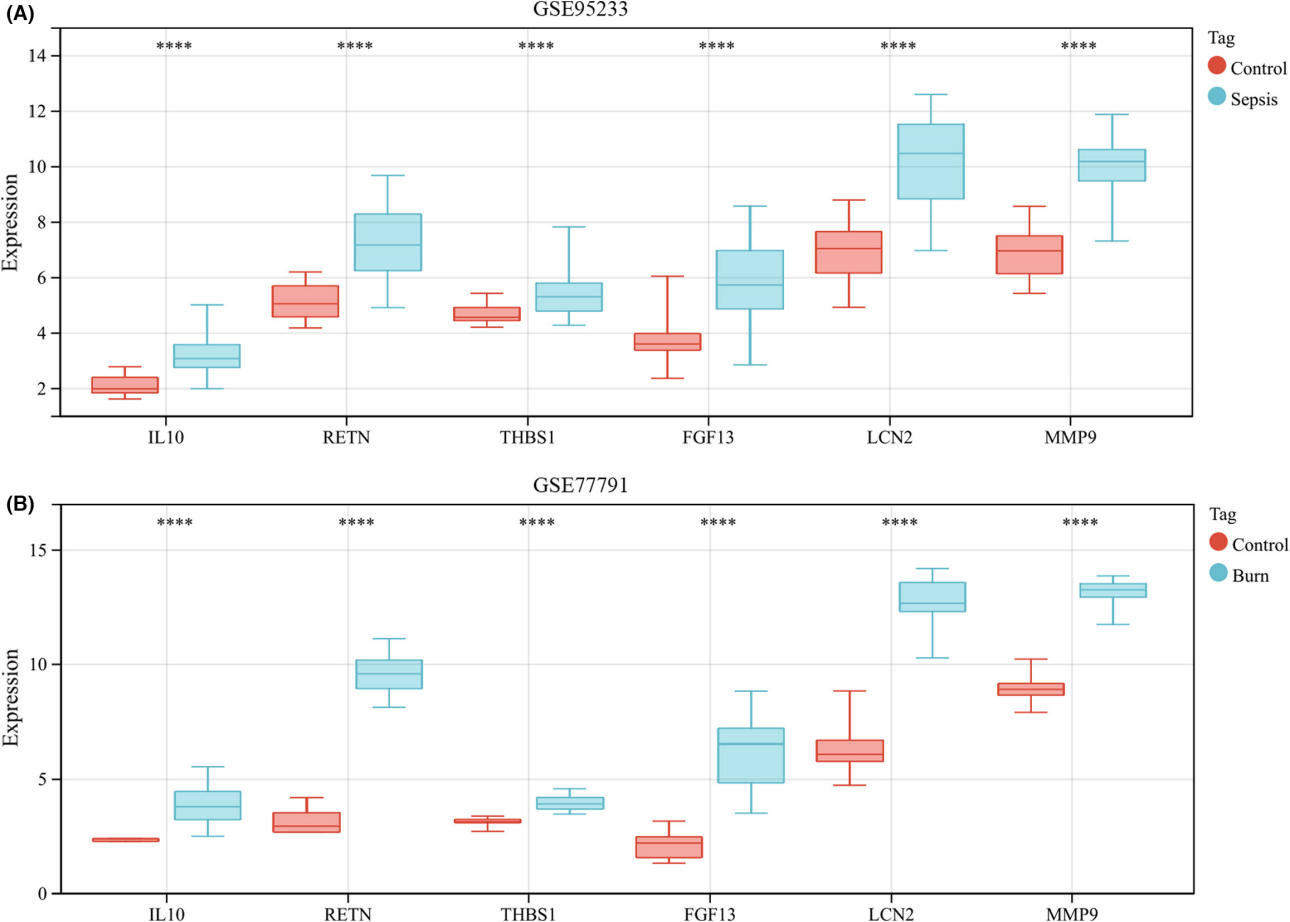
Differential expression of six core immune‐related genes in GSE95233 and GSE77791. Differences in expression of the six core genes in the original data were verified by *t*‐test. The numbers on the ordinate represent probe values. *p < 0.05; **p < 0.01; ***p < 0.001; ****p < 0.0001.

Through the STRING database, we obtained a PPI network with 205 nodes and 348 interaction pairs (Figure 5A). Subsequently, we obtained a core module containing 10 DEGs and 30 interaction pairs using the MCODE plugin (Figure 5A). After taking the intersection of the Venn diagrams, we identified six core immune‐related DEGs, namely, IL10, RETN, THBS1, FGF13, LCN2 and MMP9 (Figure 5B and Table 1). Coexpression analysis revealed that these genes showed a complex PPI network with physical interactions of 57.02%, coexpression of 29.44%, prediction of 10.34% and colocalization of 3.2% via the GeneMANIA database (Figure 5C). Finally, a *t* test confirmed that the expression of the six core immune‐related DEGs was significantly upregulated in the disease group (Figure 6).

**TABLE 1:** The details of the core immune‐related DEGs.

No.	Gene symbol	Full name	Function
1	IL10	Interleukin 10	A cytokine mainly produced by monocytes with pleiotropic effects in immune regulation and inflammation
2	RETN	Resistin	RETN is an inflammatory cytokine secreted by mouse adipocytes, named for its ability to increase insulin resistance
3	THBS1	Thrombospondin 1	This protein mediates cell–cell or cell‐matrix interactions and plays a role in platelet aggregation, angiogenesis and tumorigenesis
4	FGF13	Fibroblast growth factor 13	FGF13 is a microtubule‐stabilizing protein that regulates cytoskeletal plasticity and neuronal polarization
5	LCN2	Lipocalin 2	This protein is associated with neutrophil gelatinase and affects innate immunity by sequestering siderophores to limit bacterial growth
6	MMP9	Matrix metallopeptidase 9	This protein plays a key role in extracellular matrix protein degradation in normal physiology and in a variety of pathologies, including those associated with inflammation

### 3.5 Immune infiltration analyses

**FIGURE 7:**
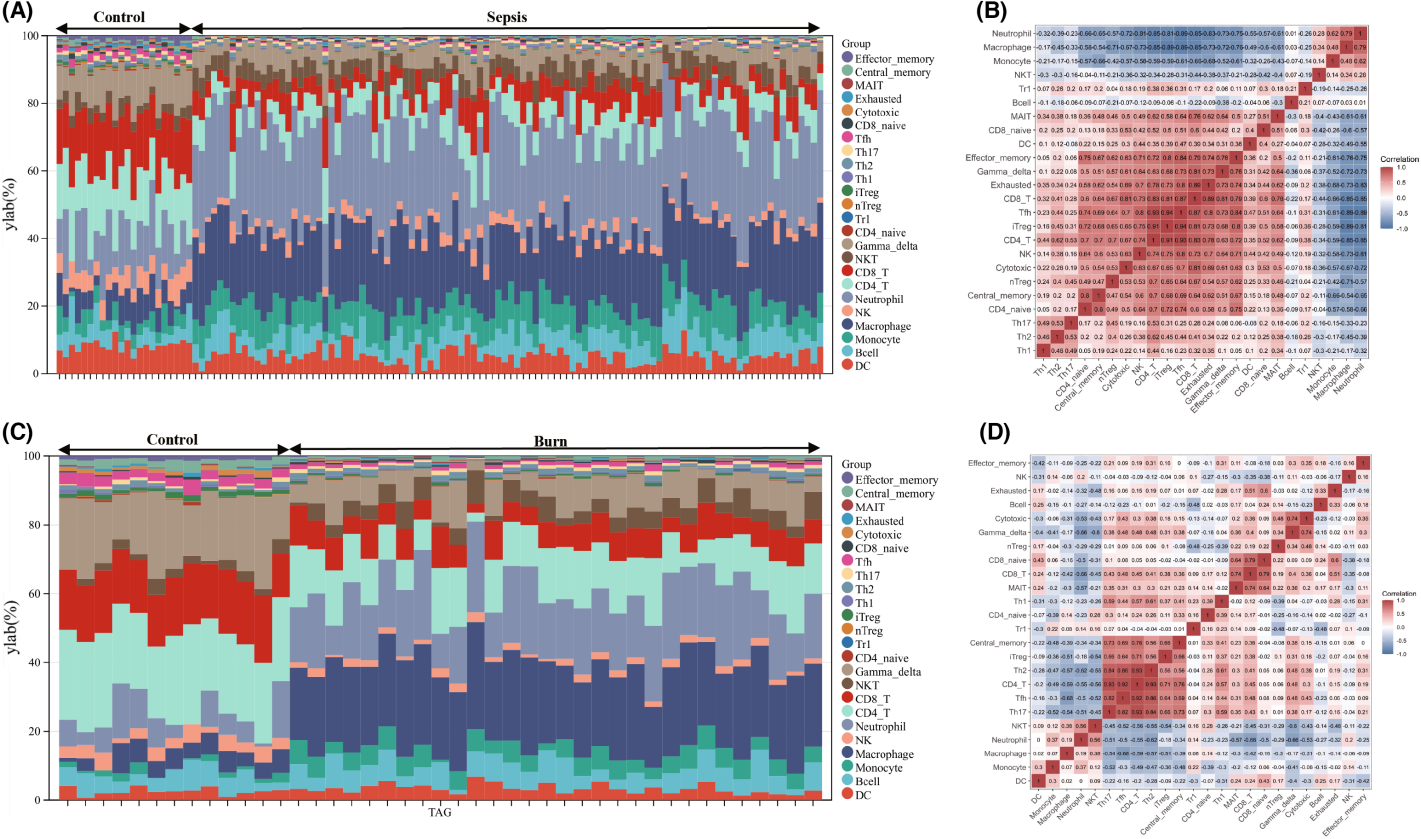
(A,C) Stacked bar chart of the immune cell. The different colours of the rectangular bars in the diagram represent different immune cells, and the length represents the proportion of immune cells. (B,D) The correlation matrix of immune cell proportions. The numbers in the squares represent the correlation coefficients between the corresponding immune cells.

**FIGURE 8:**
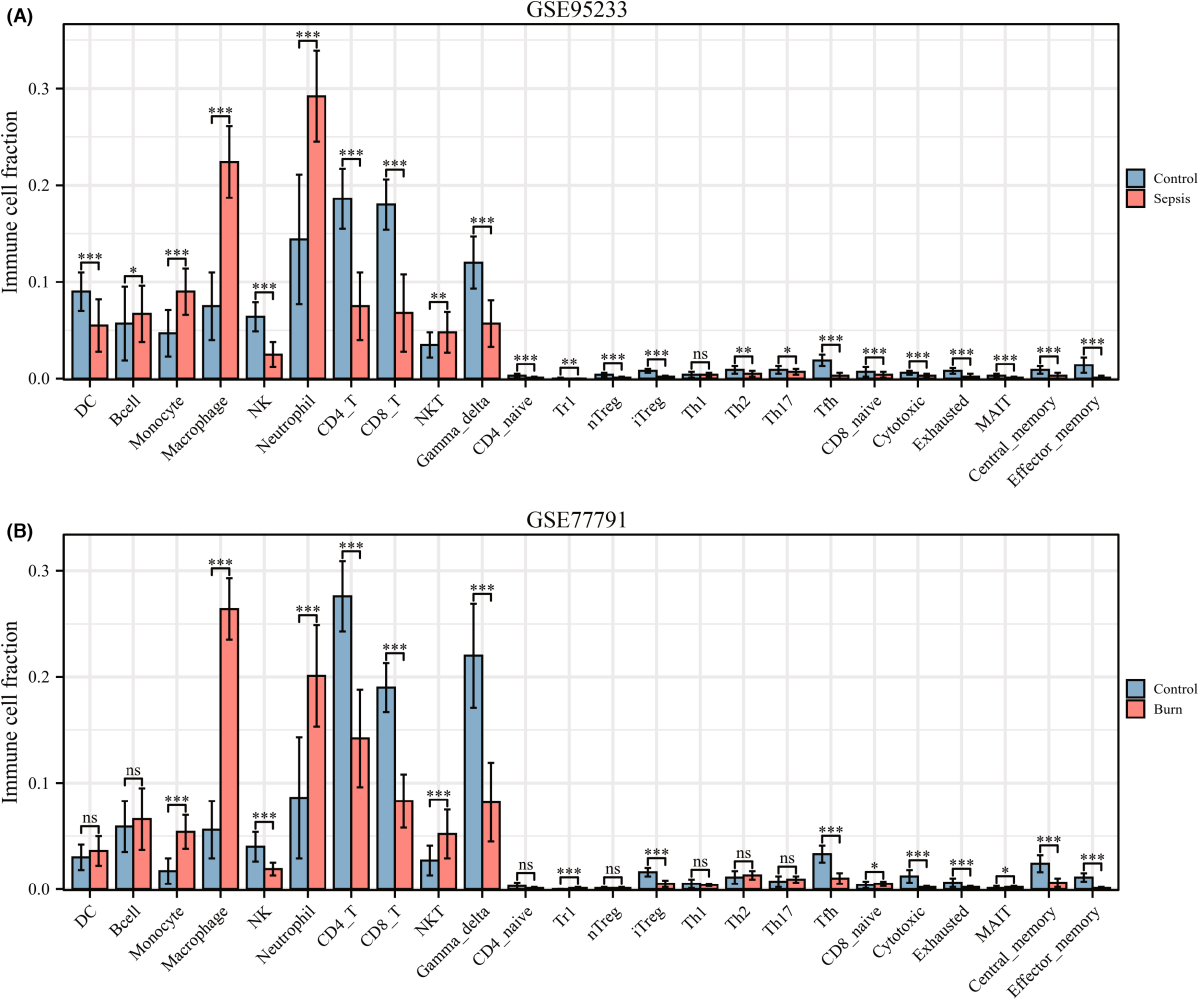
(A) Comparison of immune cell fractions between sepsis patients and healthy controls. (B) Comparison of immune cell fractions between burns patients and healthy controls. The horizontal axis represents different immune cells, and the vertical axis represents the proportion of immune cells. *t* test was used to compare the disease group and the control group. *p < 0.05; **p < 0.01; ***p < 0.001; ****p < 0.0001.

**FIGURE 9:**
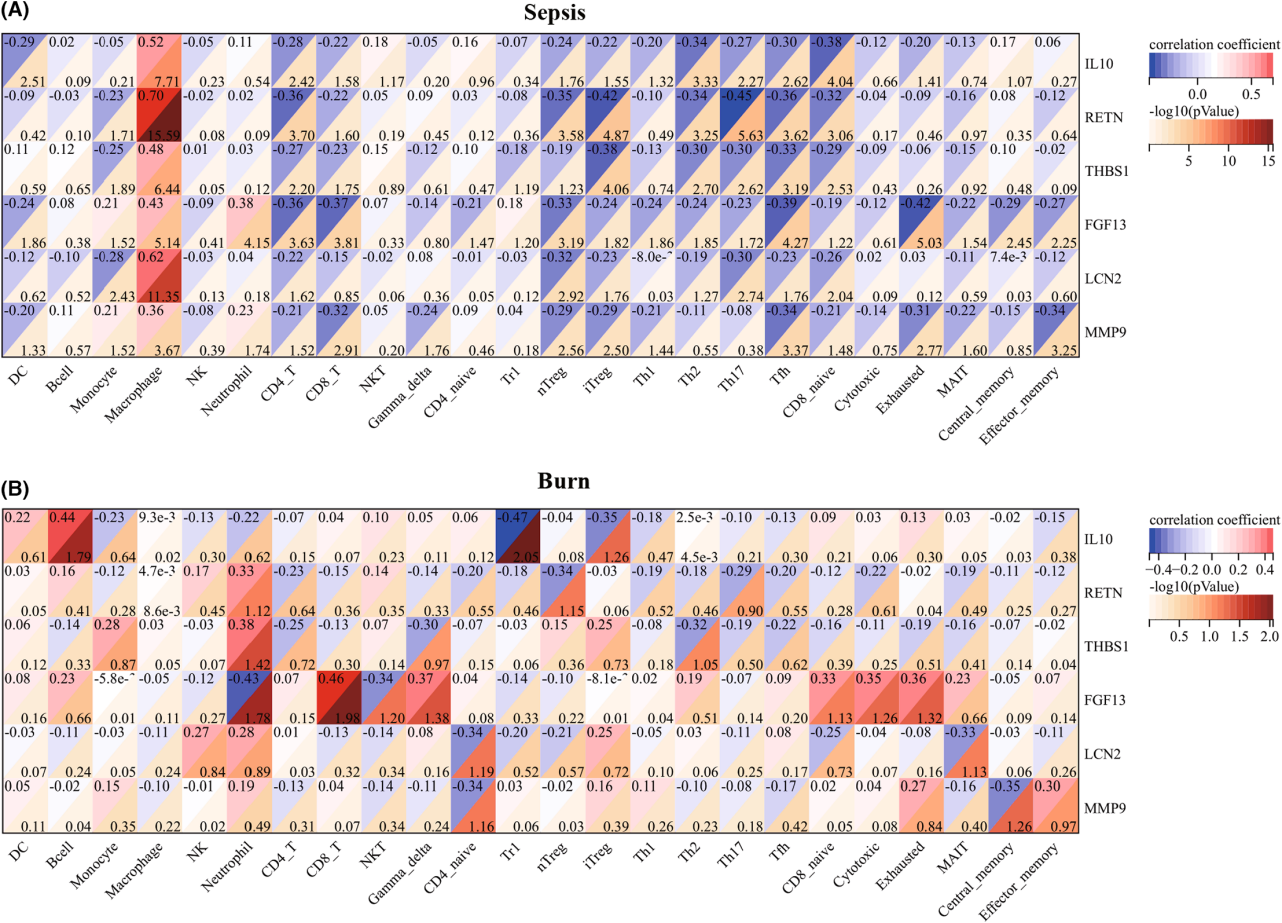
(A) Correlations between core immune‐related DEGs and immune cell components in sepsis patients. (B) Correlation between core immune‐related DEGs and immune cell components in burns patients. The number in the upper left corner of the box represents the correlation coefficient, and the number in the lower right corner represents the *p* value.

Evaluation of the immune cell composition of sepsis and burn patients indicated significant differences in immune cell profiles between the diseased and control groups (Figure 7A,C). In sepsis and burn patients, Th2 cells, Th17 cells, Tfh cells, iTregs and CD4_T cells were highly positively correlated (Figure 7B,D). In sepsis and burn patients, monocytes, macrophages, neutrophils and NKT cells were significantly increased, while NK, CD4_T, CD8_T, gamma_delta, iTreg, Tfh, cytotoxic, exhausted, central_memory and effector_memory cells were significantly decreased (Figure 8A,B). In addition, we explored associations between core immune‐related DEGs and immune cell components in sepsis and burn patients. The results showed that some core immune‐related DEGs were significantly associated with simultaneous dysregulation of immune cells in sepsis and burn patients (Figure 9A,B). For example, in sepsis patients, RETN showed a strong positive correlation with macrophages and a significant negative correlation with Th17 cells (Figure 9A). In burn patients, FGF13 was strongly positively correlated with CD8_T cells but significantly negatively correlated with neutrophils (Figure 9B).

### 3.6 Clinical relevance of core immune‐related DEGs in sepsis and burns

**FIGURE 10:**
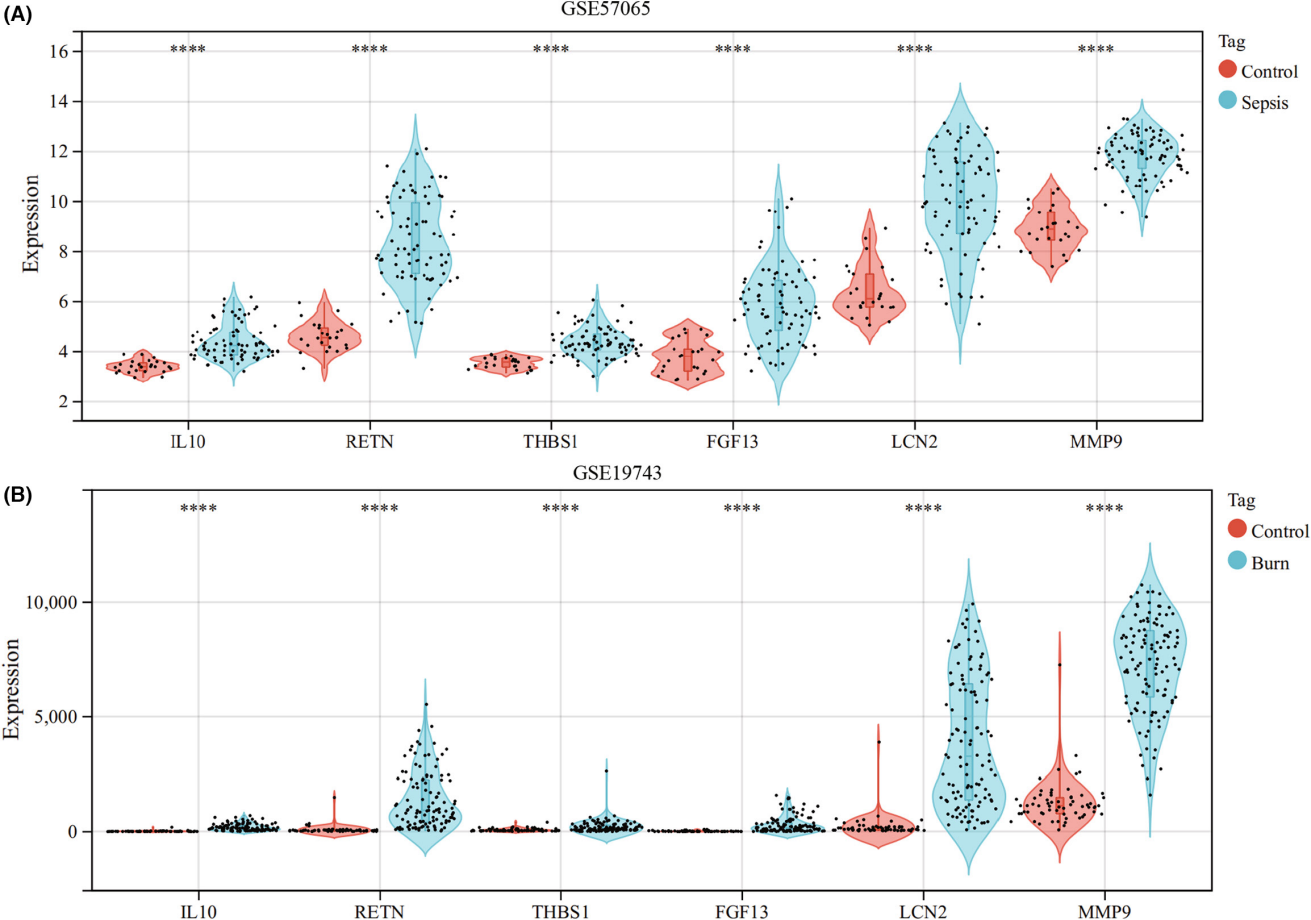
(A,B) Expression levels of core immune‐related DEGs in GSE57065 and GSE19743. The weight of violin represents the distribution density, and the numbers on the ordinate represent probe values. *p < 0.05; **p < 0.01; ***p < 0.001; ****p < 0.0001.

**FIGURE 11:**
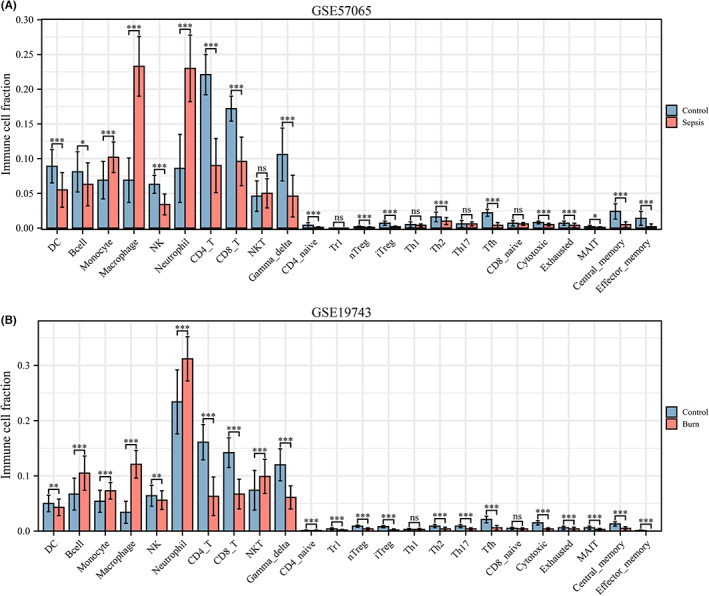
(A) Comparison of immune cell fractions between sepsis patients and healthy controls in GSE57065. (B) Comparison of immune cell fractions between burns patients and healthy controls in GSE19743. The horizontal axis represents different immune cells, and the vertical axis represents the proportion of immune cells. *t* test was used to compare the disease group and the control group. *p < 0.05; **p < 0.01; ***p < 0.001; ****p < 0.0001.

**FIGURE 12:**
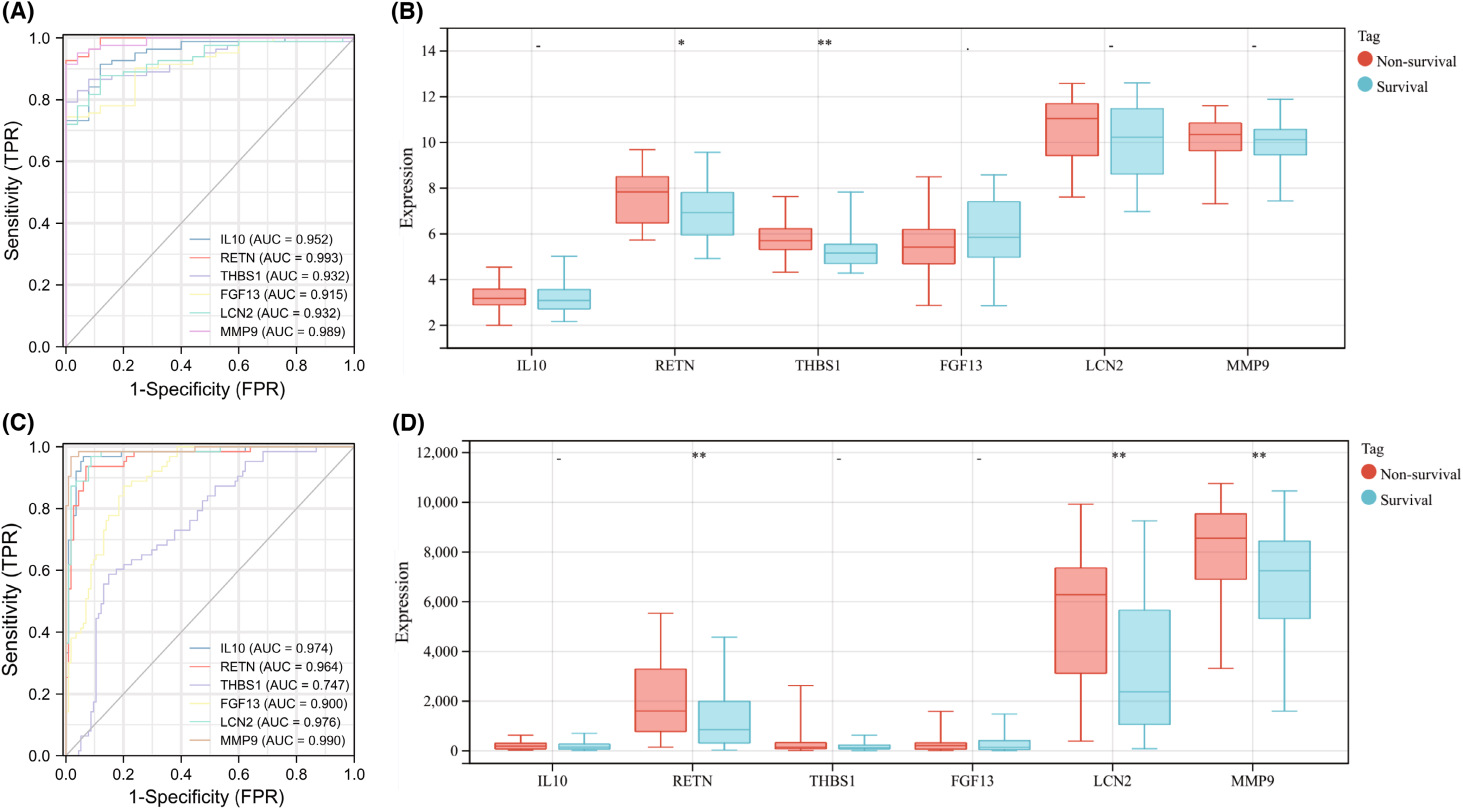
(A,C) ROC curve analysis of core immune‐related DEGs in sepsis and burns. Area under the curve (AUC) varies from 0.5 to 1.0. The closer the AUC gets to 1, the more accurate the diagnostic test is (0.5 – 0.7: low‐moderate accuracy, 0.7 – 0.9: moderate accuracy, 0.9 – 1: high accuracy). (B, D) Differences in expression of core immune‐related DEGs between survival and non‐survival of sepsis and burns patients. *t*‐test was used for analysis. *p < 0.05; **p < 0.01; ***p < 0.001; ****p < 0.0001.

According to a *t* test, the six immune‐related DEGs conformed to the expression trends in the aforementioned sepsis and burn datasets (Figure 10A,B). In addition, we analysed the immune cell infiltration profiles of the GSE57065 and GSE19743 datasets, which were consistent with previous results (Figure 11A,B). ROC analysis showed that these genes had good diagnostic value for burns and sepsis (Figure 12A,C). Because the GSE95233 and GSE19743 datasets contain survival information, we compared the differences in core immune‐related DEGs between survival and nonsurvival groups to explore the prognostic potential of these immune‐related DEGs for sepsis and burn patients. The results showed that the expression levels of RETN and THBS1 were lower in the survival group, which indicated that high expression of RETN and THBS1 was associated with a poor prognosis in the sepsis group. In the burn group, however, the expression levels of RETN, LCN2 and MMP9 were lower in the survival group, which suggested that high expression of RETN, LCN2 and MMP9 predicted a poorer prognosis (Figure 12B,D).

## 4 DISCUSSION

Burn‐induced sepsis can affect both the innate and adaptive immune systems of the body. In burn sepsis patients, the immune function of NK cells and neutrophils is impaired,^26^, ^27^, ^28^ while the phagocytic ability of macrophages is weakened.^29^ Evaluation of the immune cell composition of sepsis and burn patients identified significant differences in immune cell profiles between the diseased and control groups. In sepsis and burn patients, monocytes, macrophages, neutrophils and NKT cells were increased, whereas NK cells, CD4^+^ T cells and CD8^+^ T cells were decreased.

Due to their widespread presence in various tissues, macrophages play roles in phagocytosis, sterilization, antigen presentation and inflammatory cytokine secretion in various stages of sepsis.^30^, ^31^, ^32^ After the body suffers from burns, macrophages are overactivated into proinflammatory (M1) macrophages in the early stage of sepsis, which release inflammatory factors (IL1, IL6 and TNF‐α). Decreased phagocytic capacity has also been shown after severe burns.^33^, ^34^ Studies have shown that the release of IL4 and IL10 significantly inhibits the antigen‐presenting ability of macrophages as well as inhibits the bactericidal activity of NK cells and neutrophils.^35^, ^36^, ^37^ Therefore, there is a correlation between elevated IL10 levels and infectious events.^38^, ^39^ Interestingly, the present study identified six core immune‐related DEGs, including IL10. Correlation analysis between IL10 and immune cells in the sepsis group identified a strong positive correlation between IL10 and macrophages, providing a theoretical basis for the therapeutic effect of IL10‐modified engineered macrophages on sepsis.^40^ The JAK/STAT signalling pathway involving IL10 is an important therapeutic target in sepsis. Mesenchymal stem cells regulate The cells and inflammatory factors through the JAK/STAT signalling pathway to control the inflammatory response induced by sepsis, thus improving the survival rate of sepsis model rats.^41^ In addition, 7‐α‐obacunyl acetate significantly improves the survival rate of septic mice by inhibiting the JAK/STAT signalling pathway.^42^ Furthermore, the therapeutic effects of various microRNAs on sepsis are also achieved by regulating JAK/STAT signalling.^43^, ^44^ All the above studies indicate that the JAK/STAT signalling pathway is a potential important therapeutic target for sepsis.

In addition, the respective proportions of CD4^+^ T cells and CD8^+^ T cells decreased after severe burns.^45^ In the event of sepsis, naive CD4^+^ T cells differentiate into different cell phenotypes, namely, Th1, Th2 and Th17, after homologous antigen (Ag) expression by antigen‐presenting cells (APCs) and Tregs.^46^, ^47^, ^48^, ^49^, ^50^, ^51^, ^52^ Th1 cells activate cytotoxic CD8^+^ T cells and promote the formation of memory CD8^+^ T cells by secreting IL2.^53^, ^54^, ^55^, ^56^, ^57^, ^58^ Th17 cells are a class of effector CD4^+^ T cells capable of producing IL17, IL22 and TNF‐α, which recruit activated neutrophils.^59^ Interaction with the cognate Ag increases the number of raw Ag‐specific CD8^+^ T cells,^60^, ^61^, ^62^ and the number of Ag‐specific CD8^+^ T cells peak from days to weeks after primary infection. At this time, 95%–98% of Ag‐specific CD8^+^ T cells are eliminated in the programmed death stage, and some remaining memory CD8^+^ T‐cell populations proliferate and expand when they encounter pathogens, providing protection for the body.^62^, ^63^, ^64^ Sepsis not only affects the number of CD8^+^ T cells in patients but also alters the phenotype of CD8^+^ T cells, thereby reducing the quality of CD8^+^ T cells.^65^ Sepsis also reduces the number of immature B lymphocytes and increases the number of mature B lymphocytes and plasma cells.^66^, ^67^, ^68^ Furthermore, unlike macrophages and neutrophils, the number and function of dendritic cells decrease after sepsis.^69^, ^70^, ^71^, ^72^ Taken together, the suppression of the innate and adaptive immune responses leads to an increased incidence of infection and multiple organ failure, ultimately leading to increased mortality in burn sepsis.

Finally, we compared differences in core immune‐related DEGs between survival and nonsurvival groups to explore the impact of these immune‐related DEGs on outcomes in patients with sepsis and burns. The results showed that the expression levels of RETN and THBS1 were lower in the survival group, indicating that high expression of RETN and THBS1 is associated with poor prognosis in sepsis. In the burn group, however, the expression of RETN, LCN2 and MMP9 was lower in the survival group, suggesting that high expression of RETN, LCN2 and MMP9 predicts a poor prognosis.

RETN is an inflammatory cytokine secreted by mouse adipocytes, and it is named for its ability to increase insulin resistance.^73^ The unique role of RETN in the immune response is uncertain, but RETN increases nuclear translocation of the NF‐κB transcription factor and activation of MAPK,^74^ thereby upregulating a cascade of inflammatory mediators, such as inflammatory cytokines and adhesion molecules, as well as secretion of inducible nitric oxide synthase,^75^ Thus, RETN is considered a proinflammatory cytokine.^74^, ^76^ Furthermore, the inference that RETN is involved in the interaction among obesity, insulin resistance and low‐grade inflammation has been challenged because human adipocytes have not been demonstrated to secrete RETN and that RETN in humans is mainly secreted by monocytes.^77^, ^78^, ^79^ Recently, proteomics and RNA sequencing screening have demonstrated that RETN is highly expressed in the plasma of sepsis patients.^80^ Moreover, RETN is mainly expressed in macrophages and widely involved in inflammation and the immune response.^80^ In addition, as a key medium of immunosuppression, RETN is closely related to the morbidity and mortality of sepsis.^81^, ^82^ Stress hyperglycaemia and insulin resistance are evolutionarily conserved metabolic adaptations to severe injuries, including severe trauma, burns and haemorrhagic shock.^83^ In severely burned patients, the expression of RETN in circulation is significantly increased.^84^, ^85^, ^86^ In addition, RETN forms a network with other proinflammatory cytokines and is closely related to the severity and prognosis of severe burns.^86^ All the above studies emphasize the important role of RETN in severe burns and sepsis, thus warranting further investigation of its mechanism.

LCN2 is a secreted glycoprotein of the adipokine superfamily.^87^ LCN2, as a potential immune regulatory molecule, induces immune tolerance by upregulating the expression of human leukocyte antigen G (HLA‐G) on CD4^+^ T cells and the expansion of Tregs in healthy donors, leading to autoimmune diseases (Type 1 diabetes, systemic lupus erythematosus, rheumatoid arthritis and psoriasis).^88^ In neutrophils, LCN2 secretion is highly stimulated by inflammation and infection activation,^89^ and LPS and TNF‐α are two potent inducers of LCN2 production. Moreover, LCN2 acts as an important regulator of macrophage polarization and activator of the NF‐κB/STAT3 anti‐inflammatory pathway.^90^ In addition to its involvement in immune regulation, LCN2 interacts with MMP9 in the form of approximately 25 kDa monomers, disulfide‐linked homodimers and disulfide‐linked heterodimers.^91^, ^92^, ^93^, ^94^ The interaction of LCN2 with MMP9 is associated with the invasive behaviour of several tumour cells.^95^, ^96^, ^97^, ^98^, ^99^ Studies have shown that the expression level of LCN2 is increased in some tumour tissues, such as invasive breast cancer,^100^ pancreatic cancer^101^ and endometrial cancer,^101^ compared to normal tissues, indicating that overexpression of LCN2 is associated with the progression of a variety of cancers and is associated with poor prognosis.^102^

The present study had several limitations. The data used in the present study were obtained from public databases and lacked the support of clinical data. However, based on the future course of predicting disease trends using gene expression changes in a limited time, the present study provided some potential targets. Finally, we propose further studies on the regulatory mechanisms and functional roles of dysregulated immune‐related DEGs and immune cells to elucidate the temporal association of dysregulated immune‐related DEGs and immune cells between severe burns and sepsis.

## 5 CONCLUSIONS

In conclusion, the present study identified core immune‐related DEGs between severe burns and sepsis, including IL10, RETN, THBS1, FGF13, LCN2 and MMP9. Among these DEGs, increased expression of RETN predicts poor prognosis. Furthermore, there is some association between these core genes and dysregulated immune cells. Collectively, these dysregulated core genes and immune cells provide potential research avenues for severe burns and sepsis.

## AUTHOR CONTRIBUTIONS

**wenxing su:** Conceptualization (equal); writing – original draft (equal); writing – review and editing (equal). **Wei Li:** Data curation (equal); writing – original draft (equal); writing – review and editing (equal). **Yuanyuan Zhang:** Data curation (equal); writing – original draft (equal); writing – review and editing (equal). **Kuan Wang:** Data curation (equal); investigation (equal). **maolin chen:** Data curation (equal); investigation (equal). **Xiaoming Chen:** Data curation (equal); investigation (equal). **Dazhuang Li:** Data curation (equal); formal analysis (equal); methodology (equal); supervision (equal). **Ping Zhang:** Formal analysis (equal); investigation (equal); methodology (equal). **Daojiang Yu:** Conceptualization (equal); investigation (equal); supervision (equal).

## FUNDING INFORMATION

This work was supported by the National Natural Science Foundation of China (32071238), Natural Science Project of Chengdu Medical College (CYZYB21‐07) and Young Talent Program of China National Nuclear Corporation (CNNC2021136).

## CONFLICT OF INTEREST STATEMENT

The authors declare no conflict of interests.

## Supporting information


Table S1.

Table S2.

## Data Availability

The data analyzed in this article comes from Gene Expression Omnibus (GEO) database (http://www.ncbi.nlm.nih.gov/geo). The accession number can be found in the section 2 of the article.
